# Stroke survivor and caregiver experiences of virtual reality gaming to promote social participation: A qualitative study

**DOI:** 10.1371/journal.pone.0315826

**Published:** 2024-12-18

**Authors:** Suzanne Hoi Shan Lo, Janita Pak Chun Chau, Kai Chow Choi, Laveeza Butt, Alexander Yuk Lun Lau, Vivian Wing Yan Lee, Eddie Chi Fai Kwok, David R. Thompson

**Affiliations:** 1 Nethersole School of Nursing, The Chinese University of Hong Kong, Shatin, Hong Kong; 2 Department of Medicine and Therapeutics, The Chinese University of Hong Kong, Shatin, Hong Kong; 3 Centre for Learning Enhancement And Research, The Chinese University of Hong Kong, Shatin, Hong Kong; 4 Centre for eLearning Innovation and Technology, The Chinese University of Hong Kong, Shatin, Hong Kong; 5 School of Nursing and Midwifery, Queen’s University Belfast, Belfast, United Kingdom; Transilvania University of Brasov: Universitatea Transilvania din Brasov, ROMANIA

## Abstract

**Background:**

Virtual reality (VR) gaming is a promising technology that can be applied in stroke rehabilitation to increase survivors’ social engagement, though its optimal usage and effects on stroke recovery are not fully understood. This qualitative study aimed to investigate stroke survivors’ and caregivers’ perspectives of VR-based gaming rehabilitation modules for supporting post-stroke recovery and social participation.

**Methods:**

Twenty-eight participants (18 stroke survivors and 10 caregivers) were recruited through purposive sampling from acute hospitals in Hong Kong. Two rounds of semi-structured interviews were carried out, with the first round exploring participants’ previous knowledge of VR, views about its relevance to stroke rehabilitation, and expected benefits. The second round of interviews was conducted immediately post-intervention to investigate participants’ experience, satisfaction, and areas for improvement. Resulting data were thematically analysed.

**Results:**

Most participants were female (75%) with secondary education or above (82%). For stroke survivors, the average duration since stroke was 9.39 (SD = 10.48) years and most were first-time survivors (89%). Main themes identified included (1) Shift in attitudes towards VR technology; (2) Perceptions of VR effectiveness; and (3) Practical drawbacks and design recommendations. Participants reported positive experiences with the VR-based gaming modules, including increased confidence in mobility and heightened awareness regarding outdoor safety and accessibility. Despite concerns regarding hygiene and discomfort with gaming equipment, participants found VR gaming to be engaging and conducive to their recovery.

**Conclusion:**

The VR-based gaming modules were well-received by survivors and their caregivers and considered as an appealing and useful method of post-stroke rehabilitation. Improvements in survivors’ attitudes towards VR technology, and self-observed benefits to their physical and psychosocial health, were noted. Areas for optimisation included expansion of game length and contents, options for alternative gaming equipment, and enhanced design elements.

## Introduction

Stroke is a leading cause of long-term disability, and many survivors face reduced social participation due to restricted mobility, cognitive function, or psychological health [1]. In recent years, virtual reality (VR) technology has emerged as a promising tool for stroke rehabilitation [2, 3]. Offering a unique way of engaging the brain and nervous system through common tasks such as reaching, grasping, and manipulating objects in simulated environments, VR has shown favourable results in various aspects of post-stroke recovery, with several systematic reviews reporting benefits for motor rehabilitation, daily function, and quality of life [4–7]. Moreover, in comparison with conventional training methods which may result in loss of interest or poor adherence due to repetitive techniques, the integration of gamified elements in VR can increase survivor motivation and improve treatment adherence, both of which are vital to rehabilitation effectiveness [5, 8, 9].

Survivors’ neurocognitive and psychological health may also benefit from VR-based training, with a recent systematic review and meta-analysis of 23 trials reporting VR-based therapies to be effective in supporting the recovery of memory and executive function [10, 11]. Considering these positive results and significant associations between post-stroke cognition and social participation [12–14], it is worth exploring how novel VR-based rehabilitation programmes can be designed optimally to support survivors in reengaging with the community.

In particular, user input is necessary to facilitate the development of intuitive VR programmes that meet survivors’ needs and expectations, and are easy to operate, thus enhancing appeal and usability. For instance, previous trials suggest that stroke survivors may experience dizziness or other discomfort when using specialised VR equipment, highlighting the necessity of investigating survivor experiences with VR technology [15]. The involvement of caregivers in supporting post-stroke VR rehabilitation also requires further attention. Particularly for survivors with more severe motor or cognitive limitations, caregiver support during post-stroke recovery is essential, with evidence indicating that caregiver-mediated rehabilitation positively impacts survivors’ physical safety, functional independence, and intervention efficacy [16, 17]. As such, this study investigated the views of prospective users–stroke survivors and caregivers—in order to determine the optimal use of VR technology and guide its application in stroke rehabilitation.

## Methods

### Design

This qualitative study aimed to investigate potential users’ views and experience of a prototype game-based VR stroke rehabilitation intervention. It was embedded in a larger randomised controlled trial aimed at evaluating the effects of the game-based VR intervention on the health outcomes of stroke survivors. The results of this qualitative study will inform the refinement of the prototype for further evaluation.

### Participants and settings

Participants were recruited through purposive sampling from the stroke units of three acute hospitals in Hong Kong from 1 November 2021 to 31 May 2022. Stroke survivors and their caregivers were approached to join the study as pairs. Inclusion criteria for stroke survivors were as follows: (1) aged ≥ 18 years; (2) diagnosed with ischaemic or haemorrhagic stroke; (3) had impaired physical function after stroke with a modified Rankin Scale score of 2 to 4 and requiring community-based rehabilitative support; (4) able to sit with support; (5) have a Montreal Cognitive Assessment score above the second percentile; (6) literate in Chinese; (7) living in the community upon discharge from the hospital; and (8) had no prior experience of VR technology. Inclusion criteria for caregivers were as follows: (1) providing survivors with majority of care, and (2) literate in Chinese. The sample size was determined through the principle of data saturation, the point at which no novel themes emerged from newly-collected data [18].

Survivors with (1) a mental condition such as depression or schizophrenia; (2) a history of vestibular deficits or severe visual impairments; (3) seizure activity in the past 6 months; or (4) difficulty following instructions, were excluded from the study.

### Overview of the VR intervention

Three game-based VR training modules were designed by the project team to improve stroke survivors’ social participation (S1 Table). Each module focused on a different aspect of recovery to support survivors’ social engagement by enhancing their mobility, activities of daily living skills, and psychosocial health. Games were further tailored to target various elements of social participation, such as communication, socialisation, and outdoor activities. A commercial off-the-shelf head-mounted VR system (HTC Vive) including a headset and haptic handheld controllers was used to provide fully-immersive virtual 3D room-scale tracking and interactive experience in the virtual environment.

### Data collection procedures

Data were collected in 2 phases from January to October 2022 through semi-structured individual interviews conducted by 2 trained research assistants, while a third conducted the VR sessions. All research assistants were experienced in stroke rehabilitation research and were not involved in participants’ prior formal rehabilitation regimens. They had received training on research integrity and interviewing skills from the first author (SHSL) before conducting the interviews. The first author (SHSL) is a registered nurse and Ph.D. degree holder in health with over 10 years of experience in conducting stroke research studies, qualitative interviews, and thematic analyses. Interviews were conducted in a private room at a rehabilitation centre in each hospital, with each lasting approximately 45 minutes to one hour. In Phase 1, each stroke survivor and caregiver attended an interview separately in which an explanation of the study purpose, a brief introduction to VR and the game-based training modules were provided. Participants were then asked to share their expectations regarding the VR-based rehabilitation intervention, anticipated benefits to their recovery, and any relevant needs and concerns.

In Phase 2, survivors and caregivers were invited to experience the three VR-based training modules separately in another private room of the rehabilitation centre. Participants were guided by a research assistant and attempted each module for a period of 30 minutes, with the entire intervention lasting around 90 minutes in total (with 10-minute breaks in between). After completing all modules, a second interview was conducted immediately post-intervention with each participant inquiring about their experience, satisfaction, and recommendations for improvement. Interviews in both phases were conducted using a semi-structured interview guide with open-ended questions and prompts (S2 Table). All interviews were audio-recorded with consent and transcribed verbatim for analysis.

### Data analysis

An inductive thematic analysis approach was adopted in which we derived and identified themes and subthemes from the data with no preconceptions. This approach allowed for more flexibility in guiding our analyses to identify the patterns, themes, and concepts emerged from the interview data [19]. We analysed the phase one interviews and phase two interviews (before and after experiencing the virtual reality intervention respectively) separately, and then together, to identify any changes in participants’ perceptions of VR-based training and determine their satisfaction with the usage experience. Two research team members (SHSL and JPCC) listened to the audio files and read the transcripts multiple times to facilitate overall conceptualisation and identify preliminary patterns. Based on the initial transcript review, research questions, and interview guides, a group of tentative themes and sub-themes was determined. Transcripts were then coded independently by the two team members who met regularly to compare their coding schemes and resolve any discrepancies, with any new emerging codes being discussed and agreed upon by both members. Finalised codes were then jointly organised into the determined themes and subthemes, which were also modified as needed to better reflect participants’ experiences and meanings, and discussed and revised subsequently within the larger research group until definition and naming of the final themes were achieved. Selected verbatim passages were translated from original Traditional Chinese to English by one research assistant, and then back translated to Traditional Chinese by another research assistant. The first author (SHSL) who is fluent in both Traditional Chinese and English compared the two versions of the Traditional Chinese and modified the English translation to ensure semantic equivalence.

Strategies encouraging reflexivity were adopted to maintain the rigour and trustworthiness of the analysis [20]. During data collection, the research assistants kept reflexive journals to record participant feedback and identify any issues that might impact data analysis. Regular meetings were held with the research team in which journal notes were discussed with regards to data interpretation. Discussions were also held on the coding system and whether codes were appropriately organised into subthemes and themes to best represent the meanings and patterns of the data and address the research question. The decision-making process and authors’ reflections leading to the development of the final themes were also documented using a transparent audit trail recording the thematic analysis process. Participants were also given the opportunity to review the typed transcripts of both their interviews and provide feedback and clarifications to ensure that their experiences and perspectives were accurately captured.

### Ethical considerations

Ethical approval for this study was received from the Joint Chinese University of Hong Kong-New Territories East Cluster Clinical Research Ethics Committee (CREC Ref. no.: 2019.676). All participants provided written informed consent and were made aware of their right to refuse participation at any point without negative consequences prior to the study.

## Results

### Participants

We approached 20 stroke survivors to join the study, 10 of whom successfully invited their caregivers to participate. As two survivors were excluded due to lack of time to attend the pre-interview, a total of 28 participants comprising 18 stroke survivors and 10 caregivers took part in the study.

Most participants (Table 1 and S3 Table) were female (75%) and had received secondary education or above (82%). Survivors had ischaemic (72%) or haemorrhagic (28%) stroke; most first stroke (89%). The mean duration after stroke onset was 9.4 (SD = 10.5) years. Most survivors required assistive aids, mainly a walking stick/cane (56%) or wheelchair (28%). Four participants had communication difficulties (slower in speaking). None of the participants had previously used head-mounted VR gaming.

**Table 1:** Characteristics of the participants.

		Stroke survivors (n = 18)	Caregivers (n = 10)
*Socio-demographic characteristics*			
Age (years) ^†^		59.2 (8.1)	57.5 (8.2)
Level of education			
Primary		2 (11%)	2 (20%)
Secondary		15 (83%)	8 (80%)
Tertiary		1 (6%)	0 (0%)
*Clinical Characteristics*			
First or recurrent stroke			
First		16 (89%)	N/A
Recurrent		2 (11%)	
Assistive aids			
Unaided		3 (17%)	10 (100%)
Walking stick		7 (38%)	0 (0%)
Walking cane		3 (17%)	0 (0%)
Wheelchair		5 (28%)	0 (0%)
Type of stroke			
Haemorrhagic		5 (28%)	N/A
Ischaemic		13 (72%)	
Affected side			
Left		4 (22%)	N/A
Right		14 (78%)	

Data marked with ^†^ are presented as mean (standard deviation), all others are presented as frequency (%).

### Thematic findings

Three main themes and seven sub-themes emerged from the data (Table 2). Representative quotations from participants were selected to illustrate the themes, and are presented in Table 3. Participants were referred to by identifiers from “P01” to “P28” and their age.

**Table 2:** Main themes and sub-themes identified.

Main Themes	Sub-themes	Codes
**Theme 1: Shift in attitudes towards VR technology**	• Initial doubts of relevance to social participation post-stroke • Acceptance of VR technologys	• Minimal knowledge about VR • Aware of the increasing popularity of VR • More benefits than traditional manual-based rehabilitation • Open to trying something new
**Theme 2: Perceptions of VR effectiveness**	• Physical and cognitive benefits • Builds confidence for social participation and outdoor activities • Encourages goal-setting and self-monitoring	• Motivated to perform repetitive exercise • Promotes memory and logical thinking • A safe environment for practice • Highlighting safety tips for moving around in the real world • Allows for repeated attempts • Able to review progress across sessions • Scores of games for objective assessment • Motivated to achieve higher score than previous
**Theme 3: Practical drawbacks and design recommendations**	• Equipment-associated discomfort • Expansion of game contents and design features	• Issue of hygiene • Fear/feelings of dizziness • Discomfort arising from using headset • Unable to hold hand controller with weaker arm • More real-life scenarios • Adding voiceovers • Personalised pre-introduction to navigate the game

**Table 3:** Illustrative quotes from participants.

Main themes and sub-themes	Participant quotes
**Theme 1: Shift in attitudes towards VR technology**	
*Initial doubts of relevance to social participation post-stroke*	*“Since one of survivors’ basic needs is to carry out self-care*, *if [VR] could help improve this*, *they might gain self-confidence*, *and this would encourage them to expand their social interactions*.*”* (P25: caregiver aged 60)
	“*I think it’s very uni-directional… after all*, *it’s just you in that environment*, *you might receive some instructions or guidance but there’s no way for you to give a response*.” (P02: stroke survivor aged 49)
*Acceptance of VR technology*	“*The VR experience was really enjoyable*. *I liked the happy atmosphere*. *It made me feel like I was in a different world*, *and I could try exciting games in a safe environment*.” (P18: stroke survivor aged 53)
	“*I liked how the VR experience was interactive*. *It engages people and motivates them to keep going*.” (P20: caregiver aged 51)
**Theme 2: Perceptions of VR effectiveness**	
*Physical and cognitive benefits*	“*The VR experience helped me with my grip strength*. *I felt like I was able to hold onto things better after using it*, *like being able to use a knife to cut food*… [it] *really helped with my hands’ flexibility*.” (P11: stroke survivor aged 61)
	*“[VR] can inspire [survivors] because though they may not be able to hold a knife in real life*, *it is possible in the virtual world*. *It gives them confidence because they can do it without the fear of cutting themselves or breaking something*. *They are more willing to try and it helps to restore their awareness*.* *.* *.” (P23: caregiver aged 60)
*Builds confidence for social participation and outdoor activities*	“*The guide was really helpful because it showed me what to expect and how to handle different situations… I liked that the guide had step-by-step instructions*, *which made it easier to understand*.” (P10: stroke survivor aged 59)
	“*I was really scared to go outside after my stroke*, *but the VR training helped me practice and feel more confident… I was nervous about taking the subway*, *but it made me feel like I had already done it before*, *so I knew I could do it in real life*.” (P01: stroke survivor aged 53)
*Encourages goal-setting and self-monitoring*	“*I like that the module has different levels*. *It helped me set goals for myself and work towards achieving them*. *I felt a sense of accomplishment when I completed a task that I couldn’t do before*.” (P15: stroke survivor aged 66)
	“*I liked seeing my progress report…*. *It showed me how far I had come since I started*, *and that was really motivating*.” (P13: stroke survivor aged 67)
**Theme 3: Practical drawbacks and design recommendations**	
*Equipment-associated discomfort*	“*The helmet was tight and heavy… I could feel the physical pressure when wearing it for a long time*. *I was sweating…*.” (P06: stroke survivor aged 77)
	“*Being able to control the hand controller was really hard for me*. *Since my good hand was also holding a controller*, *I couldn’t use it to support my affected hand…*.” (P10: stroke survivor aged 59)
*Expansion of game contents and design features*	“*I liked that we were able to go through different scenarios*. *It kept things interesting*.* *.* *. *It’d be better if more scenarios could be added*, *such as demonstrating how to go to the clinic alone*.” (P09: stroke survivor aged 65)
	*“[The facilitators] could give us a briefing about what to expect first*, *like explaining that we will be entering a “kitchen” setting for example*. *This would allow us to be mentally prepared*.*”* (P11: stroke survivor aged 61)

### Theme 1: Shift in attitudes towards VR technology

#### Initial doubts of relevance to social participation post-stroke

Although most participants had heard about VR and knew it required special equipment, it was mostly within the context of gaming (for leisure) and considered to be a novel technology. Regarding the use of VR for stroke rehabilitation, while most participants were positive about its value in supporting physical training, only a minority felt that it could enhance survivors’ social participation, with some expressing reservations as they did not consider the technology adequately-equipped to facilitate interpersonal interactions.

#### Acceptance of VR technology

Despite their reservations, survivors were willing to attempt VR-based training in an effort to expand their rehabilitation options and expressed generally positive feedback regarding the trial VR experience, finding it to be exciting and attractive. Participants felt that the technology improved their overall enjoyment of physical training, suggesting higher potential engagement and adherence. The non-conventional approach of VR training was also appreciated by caregivers, who viewed it as a more stimulating experience compared with traditional rehabilitation methods.

### Theme 2: Perceptions of VR effectiveness

#### Physical and cognitive benefits

The physical training component was found to benefit participants’ motor rehabilitation as survivors reported improvements in range of motion, strength, and coordination in affected limbs. Specifically, participants noted how the VR training was able to improve essential motor abilities required for daily tasks such as gripping items.

Moreover, several participants noted that their cognitive skills were also exercised sufficiently, with benefits reported in areas such as memory, reaction time, and executive function. Heightened confidence in resuming risky daily tasks, such as handling a knife for cutting food, was also noted.

#### Builds confidence for social participation and outdoor activities

The module on outdoor safety and accessibility was most valued by participants as they remarked feeling supported and encouraged by the scenarios involving guidance on mobility and transportation. The psychosocial benefits of the experience were emphasised as participants felt less fearful and anxious about re-engaging in life situations and resuming outdoor activities.

In particular, the outdoor scenarios accompanied by instruction guides were appreciated for creating a safe and supportive environment for participants to practice and receive assurance, helping them to get accustomed to daily activities and increase their sense of self-efficacy.

#### Encourages goal-setting and self-monitoring

Participants appreciated being able to set personal goals and receive scores on their performance, which motivated them to continue practising. Moreover, the different levels allowed them to work on their specific needs and attain a sense of accomplishment when they achieved their goals. The self-monitoring feature, which included personalised progress reports, was also identified as a great source of motivation as it allowed users to track their progress over time. The competitive aspect of the training was also enjoyed, as it pushed participants to challenge themselves to do better than their peers.

### Theme 3: Practical drawbacks and design recommendations

#### Equipment-associated discomfort

In the current study, participants used a VR headset and hand-tracking controllers in both hands. Lighter equipment was recommended as the headset was found to be relatively heavy and caused discomfort such as dizziness and pressure for a few participants, which may affect training adherence and effectiveness. Hygiene concerns regarding the shared used of the VR headset were also raised.

The use of handheld controllers was found to be acceptable overall, though hemiplegic participants expressed concerns regarding their ability to grip the controllers for extended periods of time, thus necessitating a controller-free experience for users with restricted upper limb mobility. Some participants also feared bumping into surrounding objects, highlighting the need for a more spacious environment.

#### Expansion of game content and design features

The variety of scenarios included in the game were well-appreciated as they were found to help stroke survivors develop a range of essential skills and knowledge for managing daily life situations, such as slicing vegetables or using a wheelchair outdoors. It was thus suggested that the range of scenarios be further expanded to include a wider selection of situations relevant to the unique needs of participants, such as visiting health clinics. Additional levels were also suggested to lengthen gameplay and facilitate survivors in making long-term physical training plans with the VR programme.

Audio elements such as instruction guides for outdoor scenarios were also appreciated as they helped to enhance users’ comprehension of the training content. To create a more immersive experience, supplemental design features such as background music and sound effects (during movements, for example) were recommended. Arranging a short briefing prior to the VR training was also proposed to increase participants’ familiarity with the system and to prepare for the activities in the session.

## Discussion

Our findings demonstrate that the VR intervention was welcomed by the stroke participants, with perceived benefits for mobility, psychosocial health, and the resumption of social participation after stroke. However, while the stroke survivors and caregivers were largely accepting of the VR intervention as a rehabilitation strategy, modifications such as content expansion, more disability-inclusive equipment, and enhanced safety are required to address survivor concerns and optimise the user experience.

Regarding the merits of VR training in supporting survivors to join outdoor and social activities, participants noted relevant physical and psychosocial benefits and expressed higher motivation to persist with rehabilitation due to the engaging nature of the technology, as compared with traditional methods of rehabilitation which are often found to be monotonous and tedious [21]. In particular, survivors noted improvements in key movements required for daily activities such as hand flexibility and gripping, reflecting results from a recent systematic review of 24 studies which found game-based VR interventions to have positive effects on upper limb rehabilitation and hand dexterity specifically [22]. Moreover, studies show that exergaming, which involves the use of VR to conduct exercise through video gaming, also has benefits for cognitive functioning [23], which is in line with our findings. Particularly when combined with the simulation of walking aids and outdoor scenarios, survivors felt reassured and well-informed enough to attempt the same activities, demonstrating the value of guided simulations and physical training in motivating survivors to increase their social participation.

Notably, our results show that VR can indeed have a positive impact on survivors’ confidence and self-efficacy, which are crucial factors affecting participation after stroke [24]. As the virtual simulations allowed participants to practise basic motor functions in a safe and controlled environment, survivors gained confidence to re-engage in daily life situations and outdoor activities as they felt reassured by their performance in the VR training. Moreover, as the modules allowed for progress reviews, survivors gained a sense of control over their rehabilitation which encouraged them to set achievable goals and make tailored modifications to their recovery plans, thus boosting self-belief in their ability to carry out daily activities. VR training could therefore be beneficial in improving survivors’ social participation by targeting psychological recovery and strengthening survivors’ mental capacity for resuming community or outdoor activities [25].

Some concerns regarding stroke survivors’ experience of VR were also identified, one of which was the discomfort of wearing the head gear for long durations. As some stroke survivors may experience discomfort due to sensory issues, it can be challenging to wear a headset for extended periods of time. In addition, considering the reduced flexibility and mobility of those with hemiplegia, the use of hand-held controllers was also taxing. Survivors with mobility or balance issues may also find it more difficult to adapt to the dynamic movements involved in VR games and found themselves concerned about potentially bumping into nearby objects. As these issues may reduce the effectiveness of VR technology as a rehabilitation tool, it is important for healthcare professionals to address these limitations by providing stroke survivors with comfortable and suitably-fitted VR equipment alongside careful monitoring of users’ comfort levels during rehabilitation sessions.

Aside from practical issues however, the VR modules were positively appraised by both stroke survivors and caregivers for their user-friendly nature which made them a comparatively engaging method of rehabilitation. Regarding further optimisation, a pre-training briefing session was recommended to allow users some time to prepare and adapt to the system, alongside more regular progress reviews and additional daily life scenarios to provide survivors with more comprehensive and specific need-based simulations to support their community reintegration and social participation.

### Limitations

This study has some limitations. As most participants were of a younger average age for stroke survivors and had relatively low levels of post-stroke disability, the findings may not be applicable to older populations or those with more severe physical or cognitive restrictions. Studies with a more diverse population of survivors are thus required. Moreover, though survivors expressed confidence to engage in outdoor and social activities after the VR experience, their level of social participation pre- and post-VR training were not measured quantitatively. Future studies may strengthen the evidence by evaluating survivors’ social participation with validated measures.

### Implications for research and practice

Our findings provide directions for the refinement of the VR intervention and eventual programme evaluation of its effects on bolstering social participation and other health outcomes after stroke in a sufficiently-powered randomised controlled trial, and highlight a need to provide further training to healthcare professionals in facilitating the use of the VR modules. Regarding programme implementation and the optimisation of user experience, a need to provide survivors with a sense of comfort and safety emerged, which may be addressed by including a pre-training buffer period for participants to adapt to the VR equipment and system, periodical breaks to allow for rest and review, alternatives to discomfort-causing gaming equipment, enhanced hygiene measures, and a larger space to enable free and safe movement.

## Conclusion

Overall, this study found that the VR-based gaming modules were well-received by stroke survivors and their caregivers and considered to be an appealing and useful method of post-stroke rehabilitation with favourable changes in survivors’ attitudes towards VR technology, and self-observed improvements in their physical and psychosocial health. Suggested areas for optimisation included expansion of game length and contents, options for alternative gaming equipment, and enhanced design elements. Further research is required to fully evaluate the benefits of VR training in supporting post-stroke mobility and socialisation. A future randomised controlled trial should provide important data on the effectiveness of VR technology in improving social participation for stroke survivors.

## Supporting information

S1 Table Description of the three game-based VR training modules. (DOCX)

S2 Table Interview guide for semi-structured individual interviews with stroke survivors and caregivers. (DOCX)

S3 Table Characteristics of each participant. (DOCX)
