# Supplementary material for: Stroke survivor and caregiver experiences of virtual reality gaming to promote social participation: A qualitative study
Source: PLoS One. 2024 Dec 18;19(12):e0315826. doi: 10.1371/journal.pone.0315826 (PMC11654930; doi:10.1371/journal.pone.0315826)
Supplement: S1 Table — (DOCX) [file pone.0315826.s001.docx]

**Table S1. Description of the three game-based VR training modules**

| Modules | Aim | | Details |
| --- | --- | --- | --- |
| 1. Functional rehabilitation | To improve motor functioning and activities of daily living (ADL) skills of the upper limbs. | - Nine task-oriented games involving upper limb movements that progress gradually from minimal to full actions through three levels. - Activities are limited to the upper limbs and performed while sitting to minimise the risk of falls. - Handheld controllers equipped with motion sensors are used to train survivors’ fingers, wrists, and arms, encouraging the use of their weaker arm and improving post-stroke arm function. | |
| 2. Safety and accessibility outdoors | To improve independent ADL and encourage regular engagement in outdoor activities through simulation of real-life scenarios. | - Games with interactive instructions on walking aids and handling and using a wheelchair in different situations, including boarding/exiting a bus, parking, using an elevator, and general manoeuvring. | |
| 3. Social interaction and recreation | To enhance the social interaction and recreation behaviour of survivors. | - VR painting using handheld controllers, aiming to alleviate stress and anxiety and promote a sense of relaxation and calmness. - Provides opportunity for creative interactions between stroke survivors and their peers or caregivers and fosters a sense of belonging and community. | |
