# Supplementary material for: Stroke survivor and caregiver experiences of virtual reality gaming to promote social participation: A qualitative study
Source: PLoS One. 2024 Dec 18;19(12):e0315826. doi: 10.1371/journal.pone.0315826 (PMC11654930; doi:10.1371/journal.pone.0315826)
Supplement: S2 Table — (DOCX) [file pone.0315826.s002.docx]

**Table S2.** **Interview guide for semi-structured individual interviews with stroke survivors and caregivers**

| *Pre-VR experience questions* |
| --- |
| 1. What do you know about VR? (Prompts: Type, applications) |
| 1. What do you think are the most important benefits from doing VR-based rehabilitation? (Prompts: Physical, psychological, social) |
| 1. To which extent do you believe that the VR-based rehabilitation can provide you with the benefits that you mentioned? (Prompts: Elaborate with reasons) |
| 1. In your opinion, who benefits most from having a VR-based rehabilitation after discharge from the hospital? (Prompts: Level of disability, mobility) |
| 1. What conditions are needed for VR-based rehabilitation to be useful in the way you describe? (Prompts: Venue, logistics, support from healthcare professionals) |
| 1. What would you expect to feel when participating in the VR-based rehabilitation? (Prompts: Positive or negative feelings) |
| 1. What are the reasons for you to try or not to try VR-based rehabilitation? (Prompts: Level of participation, ease of use, arrangement of sessions) |
| 1. Any final thoughts or reflections on VR-based rehabilitation? |
|  |
| *Post-VR experience questions* |
| 1. How was your experience of trying the VR-based rehabilitation? (Prompts: Positive, negative experience) |
| 1. Which part(s) of the VR modules did you like most? (Prompts: Modules, games, duration) |
| 1. In your opinion, what do you think were the most important benefits from the VR-based rehabilitation? (Prompts: Physical, psychological, social) |
| 1. Did you experience any barriers or negative feelings during the experience? (Prompts: Adverse effects, dizziness) |
| 1. You mentioned that __________ was an issue for you, can you tell me more about that? (Prompts: Feelings, factors causing this) |
| 1. Do you think there is anything that could be done to help the issue? (Prompts: Arrangement of modules, equipment, type of games, session duration) |
| 1. In your opinion, what would be the features of an ideal VR-based rehabilitation game? (Prompts: Arrangement of modules, equipment, type of games, session duration) |
| 1. What are the reasons for you to try or not to receive this new VR-based rehabilitation? (Prompts: Level of participation, ease of use, arrangement of sessions) |
| 1. That is the end of the interview. Is there an aspect of your care we have not touched on, or anything you would like to add? |
