# Supplementary material for: Stroke survivor and caregiver experiences of virtual reality gaming to promote social participation: A qualitative study
Source: PLoS One. 2024 Dec 18;19(12):e0315826. doi: 10.1371/journal.pone.0315826 (PMC11654930; doi:10.1371/journal.pone.0315826)
Supplement: S3 Table — (DOCX) [file pone.0315826.s003.docx]

**Table S3. Characteristics of each participant**

| ID | Type of participant | Age (Years) | Educational level | Assistive aids used for walking | First / Recurrent stroke | Type of stroke | Affected side |
| --- | --- | --- | --- | --- | --- | --- | --- |
| P01 | Survivor | 53 | Secondary | Stick | First | H | Right |
| P02 | Survivor | 49 | Secondary | Stick | First | I | Right |
| P03 | Survivor | 47 | Secondary | Cane | First | I | Right |
| P04 | Survivor | 63 | Secondary | Cane | Recurrent | I | Right |
| P05 | Survivor | 69 | Primary | Stick | First | I | Right |
| P06 | Survivor | 77 | Secondary | Stick | Recurrent | I | Right |
| P07 | Survivor | 54 | Secondary | Wheelchair | First | I | Left |
| P08 | Survivor | 54 | Tertiary | Wheelchair | First | I | Left |
| P09 | Survivor | 65 | Primary | Wheelchair | First | I | Right |
| P10 | Survivor | 59 | Secondary | Stick | First | I | Right |
| P11 | Survivor | 61 | Secondary | Unaided | First | I | Right |
| P12 | Survivor | 61 | Secondary | Stick | First | H | Right |
| P13 | Survivor | 67 | Secondary | Wheelchair | First | H | Left |
| P14 | Survivor | 63 | Secondary | Wheelchair | First | H | Right |
| P15 | Survivor | 66 | Secondary | Stick | First | I | Right |
| P16 | Survivor | 47 | Secondary | Cane | First | H | Right |
| P17 | Survivor | 57 | Secondary | Unaided | First | I | Left |
| P18 | Survivor | 53 | Secondary | Unaided | First | I | Right |
| P19 | Caregiver | 66 | Secondary | Unaided | -- | -- | -- |
| P20 | Caregiver | 51 | Secondary | Unaided | -- | -- | -- |
| P21 | Caregiver | 75 | Primary | Unaided | -- | -- | -- |
| P22 | Caregiver | 52 | Primary | Unaided | -- | -- | -- |
| P23 | Caregiver | 60 | Secondary | Unaided | -- | -- | -- |
| P24 | Caregiver | 54 | Secondary | Unaided | -- | -- | -- |
| P25 | Caregiver | 60 | Secondary | Unaided | -- | -- | -- |
| P26 | Caregiver | 57 | Secondary | Unaided | -- | -- | -- |
| P27 | Caregiver | 53 | Secondary | Unaided | -- | -- | -- |
| P28 | Caregiver | 47 | Secondary | Unaided | -- | -- | -- |

I=Ischaemic; H=Haemorrhagic.
